# Advancing Breast Cancer Diagnosis: Optimization of Raman Spectroscopy for Urine-Based Early Detection

**DOI:** 10.3390/biomedicines13020505

**Published:** 2025-02-18

**Authors:** David Andras, Ramona G. Cozan, Delia E. Muresan, Vlad Moisoiu, George Crisan, Vasile Bintintan, George C. Dindelegan, Nicolae Leopold, Stefania D. Iancu

**Affiliations:** 1 1st Surgical Clinic, County Emergency Clinical Hospital, 400006 Cluj-Napoca, Romania; andrasdavid88@elearn.umfcluj.ro (D.A.);; 2 Department of Surgery, Iuliu Hatieganu University of Medicine and Pharmacy, 400012 Cluj-Napoca, Romania; 3 Faculty of Physics, Babeș-Bolyai University, 400084 Cluj-Napoca, Romania

**Keywords:** breast cancer, pH, SERS, liquid biopsy

## Abstract

**Background**: Surface-enhanced Raman spectroscopy (SERS) analysis of urine is a promising liquid biopsy technique for cancer detection. However, its clinical translation is hindered by two major challenges that impact classification efficacy. First, the SERS signal of urine is confounded by fluctuations induced by physiological differences in urine composition such as pH and dilution. Second, the molecular origin of the SERS signal of urine is incompletely understood, limiting the interpretability of machine learning classifiers in terms of specific biochemical markers. **Methods**: In this pilot study, we analyzed urine samples from breast cancer patients (n = 18) and control subjects (n = 10) at three pH levels (5, 7, and 9). Additionally, we analyzed simulated urine mixtures consisting of uric acid, hypoxanthine, xanthine, and creatinine in physiological concentrations to explain the variation in the SERS spectra at different pH values. **Results**: Urine at pH 9 yielded the most detailed spectral features. The SERS spectral pattern under alkaline pH reflected greater contributions from hypoxanthine, uric acid, and creatinine, while xanthine contributions diminished due to competitive interactions at the SERS substrate surface. Normalizing SERS signals to the creatinine band at 1420 cm^−1^ effectively mitigated the confounding effects of urine dilution. **Conclusions**: Optimizing the pH to 9 and normalizing to creatinine significantly enhances the interpretability and accuracy of SERS-based urine analysis for cancer detection. These findings offer important theoretical and practical advancements for the development of SERS-based liquid biopsy tools for cancer detection.

## 1. Introduction

Breast cancer is the most common cancer among women worldwide and a leading cause of cancer-related mortality. Data indicate that in Canada alone, more than 32,000 deaths have been prevented annually due to advancements in early detection and treatment [1]. Screening mammography has been shown to reduce breast cancer mortality by up to 31% when applied to women aged 40 to 74, according to a New York Health Insurance plan trial and the Swedish Two-Country trial [2,3]. Following these promising results, screening mammography has been widely adopted globally. However, despite its benefits, screening mammography is associated with certain limitations, including a high incidence of false-positive results. These occur when mammographic abnormalities are mistakenly identified as cancer, leading to patient anxiety, unnecessary testing, and biopsies. Another disadvantage is overdiagnosis, which affects approximately 11–22% of detected cases and refers to the detection of cancers that would not have caused symptoms or led to mortality within the patient’s lifetime. These types of indolent cancers may progress slowly or remain dormant, meaning they would never become clinically significant [4]. Thus, there is a need for novel screening methods that can overcome the limitations of mammography.

Surface-enhanced Raman scattering (SERS) profiling of urine is emerging as a liquid biopsy technique for breast cancer diagnosis [5,6,7,8]. SERS combines high sensitivity (comparable to fluorescence emission) with the advantages of label-free molecular detection. Its rapid analytical capability and cost-effectiveness make it particularly suitable for clinical applications [8,9,10,11]. SERS relies on the interaction of molecules with metallic nanosurfaces, resulting in the enhanced Raman signal of adsorbed species [12]. This mechanism provides high specificity, as only molecules with strong affinities for the metal surface, such as purine metabolites, are predominantly detected, while other urine metabolites have a much lower contribution to the spectral profile [9,13].

Among various biofluids, urine presents unique advantages for cancer diagnostics, including non-invasive collection, minimal pre-processing requirements, and the potential to reflect systemic metabolic changes [13]. Despite these advantages, the clinical translation of SERS-based analysis of urine has been hindered by several factors, as discussed below.

The adsorption of purine metabolites such as uric acid, hypoxanthine, and xanthine is influenced by the urinary pH [14], which ranges from 4 to 9 [15]. Given that the pKa values of purine metabolites fall within this pH range, their protonation states fluctuate, altering their affinities for the metal surface and thereby significantly affecting their SERS spectral signatures [16,17].

A second confounding factor for the SERS signal is urine dilution (commonly measured by specific gravity), which also varies widely between 1.002 and 1.037. In clinical diagnostics, normalization to creatinine concentration is typically employed to account for dilution effects, as creatinine excretion remains relatively constant throughout the day [18]. However, this normalization approach has seldom been implemented for SERS-based urine analysis.

In addition to analytical confounding factors, the clinical translation of SERS-based analysis of urine for cancer detection is also hindered by the complexity of SERS spectral interpretation. Notably, there is considerable spectral overlap among the SERS bands of purine metabolites, which complicates the clear identification of biochemical markers that ultimately enable the classification of samples via machine learning algorithms.

In this study, we investigated the effect of urinary pH on the SERS signal of urine from control subjects and patients with breast cancer, aiming to minimize spectral variability caused by pH fluctuations. We also analyzed simulated urine mixtures consisting of uric acid, hypoxanthine, xanthine, and creatinine in physiological concentrations with the aim of understanding the specific contributions of these markers to the overall SERS signal of urine at various pH values. Finally, we explored the possibility of using creatinine as an internal standard for eliminating the confounding effects of urine dilution and evaluated the impact on the classification accuracy of machine learning algorithms. By establishing a standardized approach to eliminating the confounding effects of variations in urine pH and dilution and by improving the interpretability of the SERS signal of urine, this study aims to enhance the reliability of SERS spectroscopy for cancer detection and highlight its potential as a non-invasive diagnostic tool.

## 2. Materials and Methods

### 2.1. Patient Enrolment

The study included a group of n = 18 patients who were histologically confirmed with early-stage breast cancer and referred to our hospital for elective surgery between 2023 and 2024. We also included a group of n = 10 control subjects represented by female patients who had benign screening mammography in the last 12 months. All breast cancer patients were diagnosed after breast mammography detection of malignant lesions. All patients included in the breast cancer group had proven histology of breast cancer through true-cut biopsies prior to surgery. After positive diagnosis and staging and following consensus within the hospital’s multidisciplinary team, patients underwent upfront radical surgery without neoadjuvant treatment. Surgical procedure implied lumpectomy or mastectomy with sentinel lymph node biopsy. Post-operatory histopathological assessment of the specimens was performed in order to confirm complete excision and evaluate the nodal status. Patients with large palpable masses, suspicious lymph nodes, neoadjuvant hormonal therapy, or chemotherapy were excluded. From each patient, 10 mL of urine was collected 24 h prior to the surgical procedure. Similarly, 10 mL of urine was collected from subjects in the control group that underwent screening. Urine was stored at −80 °C.

The study was approved by the Ethical Committee of the 1st Surgical Clinic, Cluj-Napoca County Emergency Clinical Hospital, and informed consent was obtained from all patients enrolled in the study.

### 2.2. SERS Spectra Acquisition

The SERS spectra were obtained using a portable Raman spectrometer (AvaRaman-532 HERO-EVO, Avantes, The Netherlands with a spectral resolution of 10 cm^−1^) connected to a Raman probe (AvaRaman-PRB-532, Avantes, The Netherlands, 7.5 mm focal length, 3 mm laser spot size) operated in the clinical setting. For each sample, three 10 s acquisitions were performed using a 532 nm laser excitation line (50 mW). No fluorescence background from the samples was observed using the 532 nm laser line. Hydroxylamine hydrochloride–reduced silver nanoparticles (hya-AgNPs) were employed as the SERS substrate [19], and calcium ions (Ca^2+^) were used to enhance the SERS spectra of urine [9]. A mixture of 45 µL of hya-AgNPs, 5 µL of urine, and 5 × 10^−3^ M Ca(NO_3_)_2_ (Merck, Darmstadt, Germany) was prepared (the final concentration of Ca^2+^ was 5 × 10^−4^ M). Subsequently, 10 µL of the mixture was placed on a microscope slide and a Raman probe was used to acquire the SERS spectra. pH adjustments were performed on 100 µL aliquots of urine samples by adding HNO_3_ (Nordic Chemicals, Cluj-Napoca, Romania) and NaOH (Nordic Chemicals, Cluj-Napoca, Romania) until the desired pH value was reached. Each spectrum represents an average of three acquisitions, with 10 s integration time each.

### 2.3. Data Analysis

All data were preprocessed using the Quasar-Orange software, version 1.9.2 (Bioinformatics Laboratory of the University of Ljubljana [20]). SERS spectra were truncated in the 500–1800 cm^−1^ range, followed by rubber band baseline correction and vector normalization. Principal component analysis (PCA) was performed, retaining the first principal components (PCs) that explained 95% of the cumulative variance. The explained variance is illustrated in Supplementary Figure S1. For the analysis of unprocessed urine samples at physiological pH values, eight PCs were retained. In contrast, for urine samples adjusted to controlled pH values, seven PCs accounted for 95% of the cumulative variance. The PCs used to depict the sample distribution were selected based on Student’s *t*-test.

The PCs explaining 95% of the cumulative variance were then used as input for the logistic regression (LR) classifier with Lasso regularization and C = 1, validated through leave-one-out cross-validation.

## 3. Results and Discussion

### 3.1. pH-Dependent SERS Spectral Variations of Urine Metabolites

**Figure 1:**
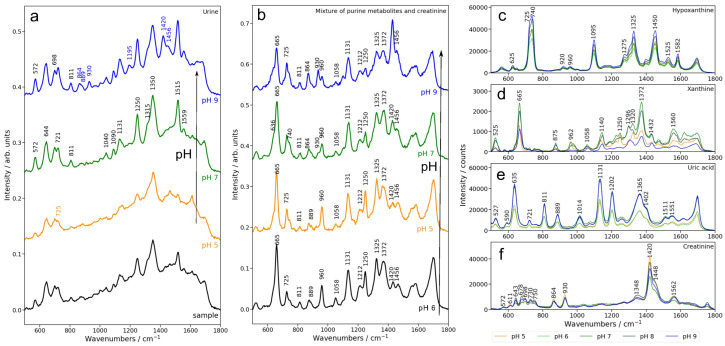
(**a**) Mean SERS spectra of urine samples from breast cancer patients at the collected pH values (black) and adjusted to pH values of 5 (orange), 7 (green), and 9 (blue). (**b**) SERS spectra of a mixture containing creatinine (10^−3^ M), uric acid (10^−4^ M), hypoxanthine (10^−5^ M), and xanthine (10^−5^ M), adjusted to pH values ranging from 5 to 9. SERS spectra of individual components adjusted to different pH values: (**c**) hypoxanthine, (**d**) xanthine, (**e**) uric acid, and (**f**) creatinine, at pH 5 (orange), pH 6 (lime green), pH 7 (green), pH 8 (dark green), and pH 9 (blue).

The adsorption of molecules from a complex matrix like urine occurs competitively, driven by their relative affinity for the metal surface, which in turn depends on pH. To explore the effect of pH, we analyzed the SERS spectra of urine from patients with breast cancer (n = 18) and control subjects (n = 10), and compared the SERS spectra of urine at their initial pH (Figure 1a, black spectrum) with the SERS spectra of urine after we adjusted all samples to pH 5, 7, and 9 (Figure 1a, orange, green, and blue spectra). The initial pH values of the urine samples ranged from 5 to 7, consistent with the normal physiological pH range of 4–9 [15]. The SERS spectra of urine at their initial pH were dominated by intense SERS bands corresponding to hypoxanthine (725, 1090, 1456 cm^−1^), while bands associated with uric acid at 811, 889, 1131, and 1515 cm^−1^ had lower-intensity. In contrast, the SERS spectra of urine adjusted to alkaline conditions (pH 9) yielded intense SERS bands across all bands.

To better understand the assignment of the SERS bands of urine, we systematically analyzed the effect of pH on the SERS signal of simulated urine represented by a mixture of the main contributors to the SERS signal (uric acid, xanthine, hypoxanthine, and creatinine) at physiologically relevant concentrations (Figure 1b). In parallel, we analyzed the SERS signals of uric acid, xanthine, hypoxanthine, and creatinine individually across the same range of pH values (Figure 1c–f).

Uric acid exhibited the strongest SERS signals at pH 9, consistent with the increased concentration of anionic uric acid in this pH range (Figure 1e). This trend is also reflected in the SERS spectra of real urine (Figure 1a) as well as in the SERS spectra of simulated urine (Figure 1b), where the intensity of the SERS bands at 635, 811, 889, and 1131 cm^−1^ attributed to uric acid increases progressively from pH 5 to pH 9.

Xanthine exhibited maximal SERS intensity at pH 7, with diminished signals under both acidic and alkaline conditions (Figure 1d). In the case of urine and simulated urine, its detection in urine samples is challenging because of significant overlap with uric acid bands, particularly at 665 and 1559 cm^−1^ (Figure 1a,b). In simulated urine, increasing the pH results in a shift from a sharp band at 665 cm^−1^, associated with xanthine, to a reduction of this band and the emergence of a shoulder as uric acid becomes more prominent in the SERS spectrum. A distinct band at 1250 cm^−1^, attributed to xanthine, is also observed, which slightly decreases with higher pH in simulated urine. In contrast, in real urine, this band slightly intensifies with increasing pH (Figure 1a).

Hypoxanthine exhibited its highest SERS intensity at pH 9 (Figure 1c), although its spectral variations between pH 5 and 9 were relatively subtle. In real and simulated urine samples, the hypoxanthine SERS bands at 725, 740, and 1090 cm^−1^ are well-defined across all pH values in this range (Figure 1a). Two distinct bands, at 725 cm^−1^ and 740 cm^−1^, were identified, both corresponding to hypoxanthine. The 725 cm^−1^ band is attributed to the keto tautomer, while the 740 cm^−1^ band represents the enolic tautomer [21]. Notably, in urine samples, the keto tautomer is predominant, which explains the absence of the 740 cm^−1^ band in the SERS spectrum of urine (Figure 1a) [22].

Creatinine showed the strongest SERS signals at pH 5, with minimal variation across the pH range (Figure 1f). However, in real and simulated urine samples, the creatinine SERS band at 1420 cm^−1^ was only observed at pH 9, while the intensity of the SERS band at 572 cm^−1^ increased with rising pH values.

In conclusion, for uric acid and hypoxanthine, the higher intensity of the SERS signal in real and simulated urine under alkaline conditions (pH 9) parallels the behavior of these metabolites when measured alone. In contrast, there is a disjunction between the effect of pH on the SERS intensity of creatinine bands in real or simulated urine on the one hand and creatinine alone: the SERS bands of creatinine are favored by alkaline conditions, but this is not the case for creatinine measured alone.

Thus, the observed variations in SERS signals are not solely attributable to the adsorption behavior of individual metabolites but are also influenced by competitive adsorption on the metal surface at different pH values. Colloidal nanoparticles offer a limited adsorption surface, which is typically saturated at analyte concentrations in the range of 10^−5^–10^−4^ M. Above this concentration the adsorption of metabolites becomes competitive, driven by their relative affinities for the silver surface. The protonation state, which varies with pH, plays a critical role in determining these affinities.

In the case of creatinine, the reduced intensity of the creatinine-associated bands in acidic conditions likely reflects its decreased adsorption affinity when competing with uric acid, hypoxanthine, and xanthine, which exhibit stronger affinities for the silver surface under these conditions. However, under alkaline conditions, despite the lower SERS intensity of individual creatinine, the competition dynamics shift, and in this environment, all metabolites, including creatinine, successfully adsorb onto the silver surface, becoming clearly detectable in the SERS spectrum.

### 3.2. Implications for Diagnostic Applications

The pH-dependent analysis of urine samples as well as of metabolites, both individually and in mixtures, revealed that pH 9 yields the most intense SERS bands, including the SERS bands of creatinine, which could be used as an internal standard to control for urine dilution. Creatinine is a convenient internal standard to control for urine dilution because the daily output in humans is relatively constant (around 1 g). Thus, we sought to explore whether bringing all urine samples to pH 9 followed by normalization to the creatinine band at 1420 cm^−1^ improves the classification accuracy.

**Figure 2:**
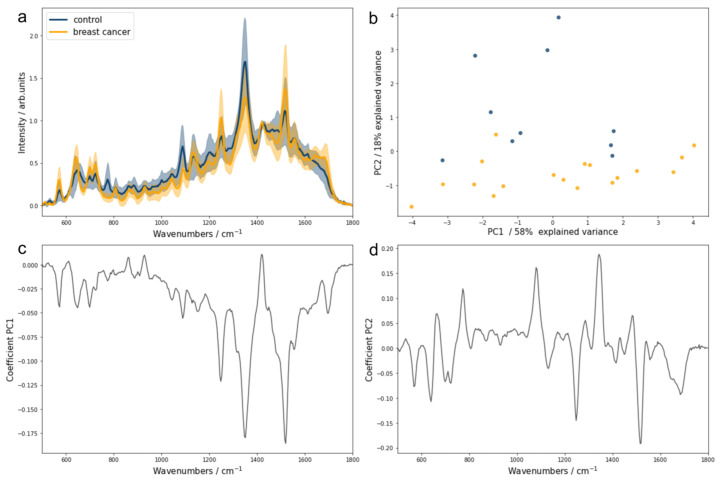
Urine SERS spectra normalized to creatinine. (**a**) Mean SERS spectra of control subjects and breast cancer patients at pH 9, normalized to the creatinine band (1420 cm^−1^), with standard deviations. (**b**) Scatter plot of principal component (PC) 1 and PC2 score values. Loading plots of PC1 (**c**) and PC2 (**d**). Control group is represented in blue color and breast cancer group in yellow.

Towards this end, we compared the results of the PCA performed on urine samples at their initial pH and after bringing the urine samples to fixed pH values. The results showed that the spectral differentiation between the control and breast cancer groups was optimal for urine samples brought to pH 5 and pH 9 (Figures S2–S6). Given that urine samples brought to pH 9 also featured a creatinine-associated SERS band suitable for normalization, we propose it as the optimal pH for SERS-based diagnostics. Figure 2 presents the normalized SERS spectra (relative to the 1420 cm^−1^ creatinine band) of urine at pH 9 (Figure 2a) and the resulting PCA scatter plot (Figure 2b), illustrating the separation of breast cancer and control groups based on PC1 and PC2 score values (Figure 2c,d).

The normalization of urine SERS spectra to the creatinine band at 1420 cm^−1^ highlighted significant differences between the control and breast cancer groups. Notably, the intensities of the SERS bands associated with hypoxanthine (725 cm^−1^) and uric acid (889 and 1131 cm^−1^) were distinct between the groups (Figure 2a). The scatter plot of PC1 and PC2 score values indicates unsupervised grouping, primarily driven by PC2 (Figure 2b). The loading plot for PC2 showed a negative contribution from SERS bands at 774, 1080, and 1334 cm^−1^, which means that samples with high PC2 score values had relatively lower intensities for these bands (Figure 2b,c). Interestingly, these bands do not seem to stem from the purine metabolites analyzed in this study. Conversely, bands associated with uric acid and xanthine were more pronounced in the breast cancer group, highlighting their potential diagnostic relevance. However, since purine metabolites are not exclusive markers for breast cancer, their role in cancer screening has also been noted in other types of cancer [9,23,24,25]. This study emphasizes the importance of optimizing SERS spectral acquisition to improve reliability for cancer detection. Further research comparing different cancer types and subtypes is needed to determine whether these markers can effectively differentiate between cancers.

Next, we investigated whether adjusting all urine samples to pH 9, followed by normalization to creatinine, would optimize classification accuracy. To assess this, the first five PCs from each pH condition were used as input for an LR classifier. The results indicated a trend of increasing classification accuracy with higher pH levels (Supplementary Table S1). Correction to pH 9 combined with normalization to the creatinine band at 1420 cm^−1^ achieved the best performance, with a classification accuracy of 85.7% (Table 1).

Additionally, PCA of the SERS spectra of urine acquired at pH 9, followed by normalization to creatinine, captured the highest proportion of cumulative variance within the first five PCs (Figure S7).

A notable limitation of this study is the small sample size, which precluded robust external validation of the LR models. However, the head-to-head comparison of PCA and LR model results provided valuable evidence supporting the critical role of pH adjustment and normalization to creatinine bands in enhancing SERS-based diagnostic tools for breast cancer. The validation of these findings in larger cohorts is warranted.

## 4. Conclusions

This study underscores the clinical potential of SERS as a non-invasive tool for breast cancer detection. By addressing key challenges such as pH variability and urine dilution, we demonstrated that urine at pH 9 yields the most detailed and diagnostically valuable spectral features. The significant contributions of metabolites like hypoxanthine, uric acid, and creatinine to the SERS signal at this optimal pH enhanced the method’s sensitivity and specificity. Normalizing the SERS spectra to the creatinine band effectively mitigates dilution effects, leading to improved classification accuracy with machine learning models. These findings pave the way for integrating SERS into clinical workflows, potentially reducing reliance on more invasive procedures like biopsies and lowering the rates of overdiagnosis and false positives in breast cancer screening. Further validation with larger, diverse patient cohorts will be essential to confirm these results and fully realize the potential of SERS-based urine analysis as a practical, cost-effective liquid biopsy tool in oncology.

## Figures and Tables

**Table 1:** Confusion matrix for the logistic regression (LR) model applied to classify control subjects and breast cancer patients based on SERS urine spectra at pH 9, normalized to creatinine.

		Predicted	
		Control	Breast Cancer
Actual	Control	8	2
	Breast cancer	2	16

## Data Availability

The original contributions presented in this study are included in the article. Further inquiries can be directed to the corresponding author.

## Supplementary Materials

The following supporting information can be downloaded at https://www.mdpi.com/article/10.3390/biomedicines13020505/s1, Figure S1: Cumulative explained variance for samples: pH 5 (B), pH 7(C), pH 9 (D); Figure S2: Mean SERS spectra of control and breast cancer patients with standard deviations at the real pH value of the samples: pH 5 (C), pH 7 (E), pH 9 (G), and the scatter plot of the significant PCs for the discrimination of the two groups at normal pH (B), pH 5 (D), pH 7 (F), and pH 9 (H). For the physiological pH, PC2 was found to be significant (*p* = 0.007). For pH 7, PC1 (*p* = 0.0002) and PC2 (*p* = 0.03) were significant. For pH 5, PC2 (*p* = 0.02) and PC5 (*p* = 0.001) were significant. For pH 9, PC1 was significant (*p* = 0.0001); Figure S3: Loading plot of principal component 1 (A) and PC 2 (B) from the PCA analysis of samples at their initial pH values; Figure S4: Loading plot of principal component 1 (A) and PC 2 (B) from the PCA analysis of samples at pH 5; Figure S5: Loading plot of principal component 1 (A), PC 2 (B), and PC7 (C) from the PCA analysis of samples at pH 7; Figure S6: Loading plot of principal component 1 (A) and PC 2 (B) from the PCA analysis of samples at pH 9; Figure S7: Cumulative explained variance for samples at pH 9 normalized to creatinine; Table S1: Classification performance of the logistic regression classifier for control and breast cancer samples, evaluated using SERS spectra of urine measured at native pH and adjusted pH levels of 5, 7, and 9.

## Abbreviations

The following abbreviations are used in this manuscript:

SERS	surface-enhanced Raman spectroscopy
hya-AgNPs	hydroxylamine hydrochloride–reduced silver nanoparticles
PCA	principal component analysis
PCs	principal components
LR	logistic regression
arb. units	arbitrary units
