# Supplementary material for: Advancing Breast Cancer Diagnosis: Optimization of Raman Spectroscopy for Urine-Based Early Detection
Source: Biomedicines. 2025 Feb 18;13(2):505. doi: 10.3390/biomedicines13020505 (PMC11852463; doi:10.3390/biomedicines13020505)
Supplement: Supplementary file 1 [file biomedicines-13-00505-s001.zip › biomedicines-3469016-supplementary-done.pdf]

biomedicines

MDPI

Supplementary Material

Advancing Breast Cancer Diagnosis: Optimization of Raman Spectroscopy for Urine-Based Early Detection

David Andras [1,2] Ramona G. Cozan [3], Delia E. Muresan [1], Vlad Moisoiu [3], George Crisan [3], Vasile Bintintan [1,2], George C. Dindelegan [1,2], Nicolae Leopold [3,*] and Stefania D. Iancu [3,*]

1 1st Surgical Clinic, County Emergency Clinical Hospital, 400006 Cluj-Napoca, Romania; andrasdavid88@elearn.umfcluj.ro (D.A.)
2 Department of Surgery, Iuliu Hatieganu University of Medicine and Pharmacy, 400012 Cluj-Napoca, Romania
3 Faculty of Physics, Babeș-Bolyai University, 400084 Cluj-Napoca, Romania
* Correspondence: nicolae.leopold@ubbcluj.ro (N.L.); stefania.iancu@ubbcluj.ro (S.D.I.)

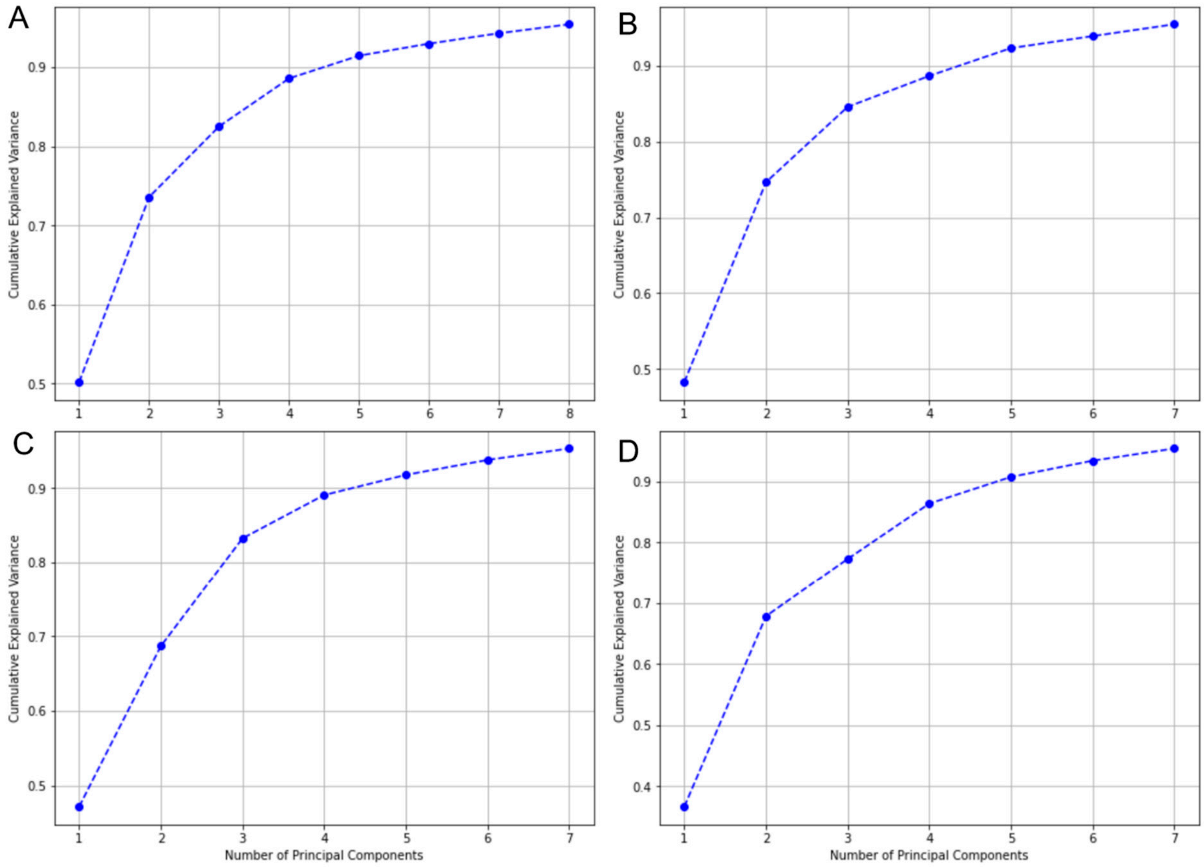

Figure S1. Cumulative explained variance for samples, pH 5 (B) pH 7(C) pH 9 (D).

Sample grouping at different pH values. The pH-dependent analysis of urine samples as well as of metabolites, both individually and in mixtures, reveals that pH 9 yields the most informative SERS spectra, consistent with the classification results discussed next.

To evaluate the grouping accuracy of urine samples, Principal Component Analysis (PCA) was performed on SERS spectra from n=18 breast cancer samples and n=10 control samples, analyzed at their physiological pH and at adjusted pH values of pH 5, 7 and 9. Figure S2 presents the mean SERS spectra for CTRL and BC samples at native and adjusted pH, along with PCA scatter plots illustrating sample grouping based on principal component (PC) 1 and PC 2 score values.

 https://doi.org/10.3390/biomedicines13020505

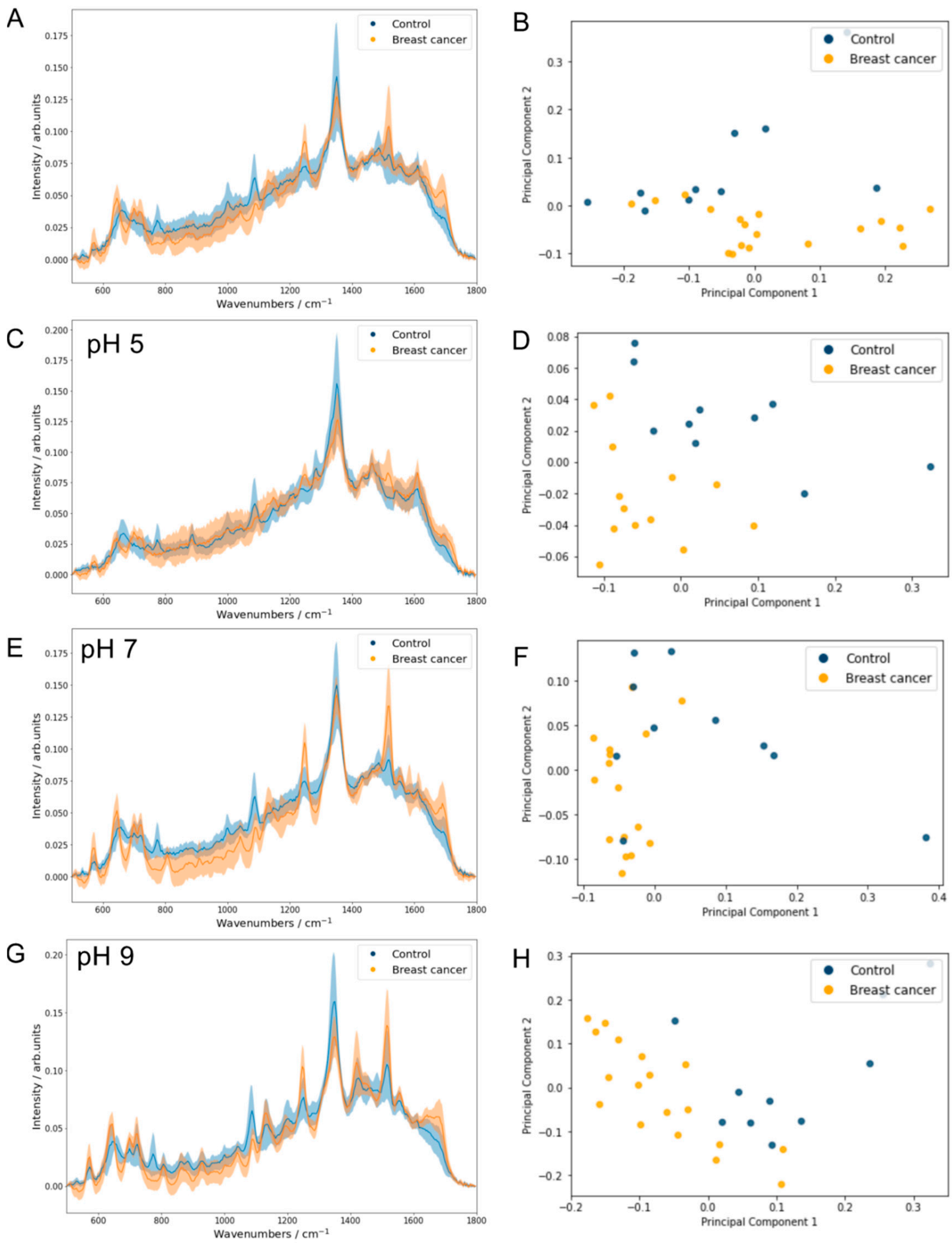

Figure S2. Mean SERS spectra of Control and Breast cancer patients with standard deviations at the real pH value of the samples: pH 5 (C), pH 7 (E), pH 9 (G), and the scatter plot of the significant PCs for the discrimination of the two groups at normal pH (B), pH 5 (D), pH 7 (F), and pH 9 (H). For the physiological pH, PC2 was found to be significant ($p = 0.007$). For pH 7, PC1 ($p = 0.0002$) and PC2 ($p = 0.03$) were significant. For pH 5, PC2 ($p = 0.02$) and PC5 ($p = 0.001$) were significant. For pH 9, PC1 was significant ($p = 0.0001$).

A visual inspection of the SERS spectra reveals significant differences between breast cancer (BC) and control (CTRL) samples across all pH values. At pH 5, the CTRL group exhibited a prominent band at 774 cm^{-1}, which was weak in BC samples. In contrast, BC spectra featured distinct bands at 640 and 720 cm^{-1}, masked in the CTRL spectra by a broad band centered at 660 cm^{-1}. Additionally, a unique band at 1515 cm^{-1} was observed

in BC spectra. PCA analysis further emphasized these distinctions, showing clustering along PC2, where the loading plot (Figure S3) indicated a negative correlation between bands at 774 cm^{-1} and 1515 cm^{-1} and those at 640, 700, and 720 cm^{-1}.

At pH 7, the 774 cm^{-1} band persisted in CTRL spectra but was absent in BC samples. The 720 cm^{-1} band appeared in both groups but with reduced intensity in the CTRL samples. A distinct BC band emerged at 1130 cm^{-1}, further distinguishing the groups.

At pH 9, the 774 cm^{-1} band remained exclusive to CTRL samples, while bands at 640, 700, and 720 cm^{-1} were present in both groups but with varying intensities, demonstrating the pH-dependent nature of spectral differences. PCA analysis at this pH identified the creatinine-associated band at 1420 cm^{-1} as a key differentiator in the PC1 loading plot. The signal-to-noise ratio was optimal at pH 9, making it the most informative condition.

The clustering of CTRL and BC groups across all pH values was driven by the negative correlation between bands at 774, 1083, and 1515 cm^{-1} and those at 640, 700, and 720 cm^{-1}, as shown in the PC2 loading plots for pH 5, 7, and native pH (Figures S3, S4, and S5). At pH 9, however, the enhanced creatinine signal at 1420 cm^{-1} emerged as the most relevant distinguishing feature, supporting its role in separating the two groups (Figure S6).

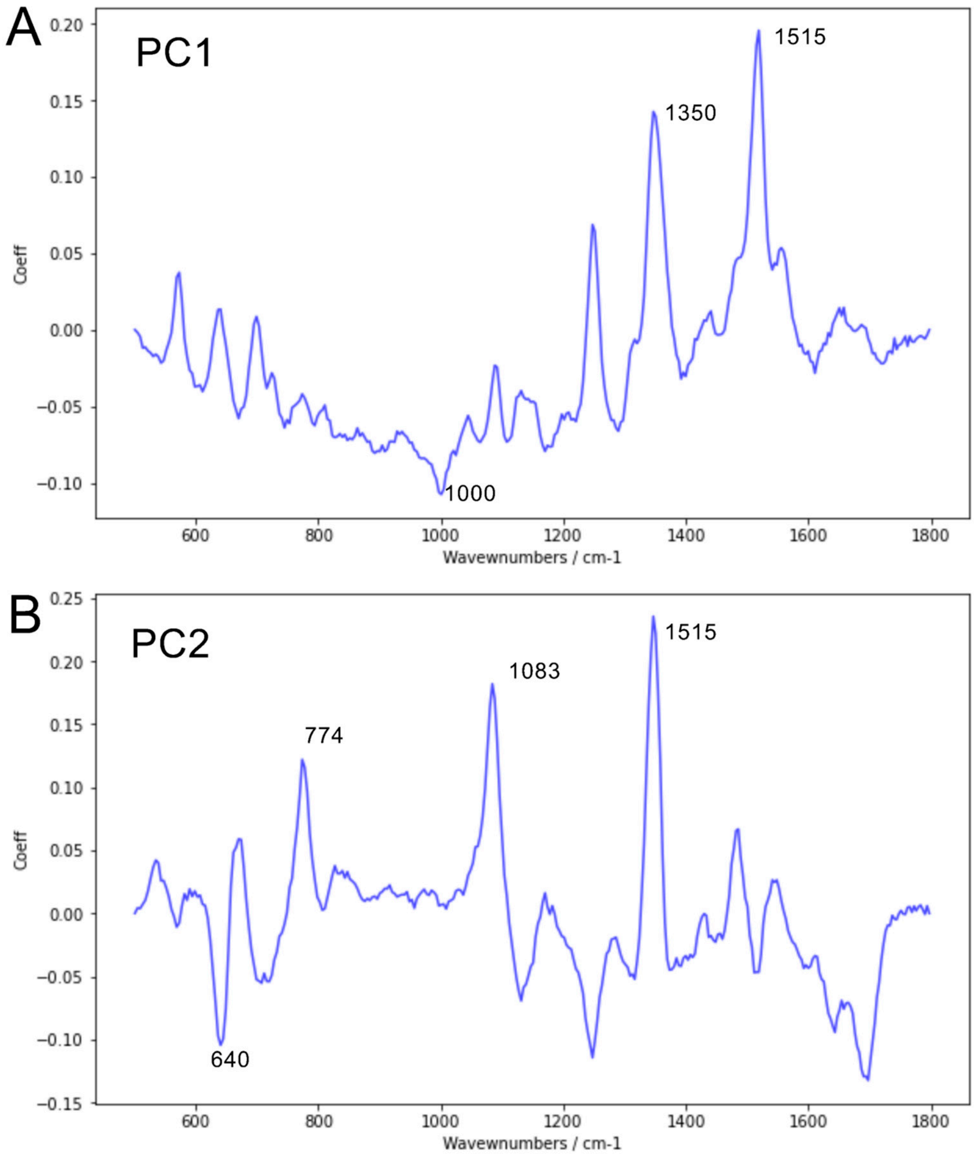

Figure S3. Loading plot of Principal Component 1 (A) and PC 2 (B) from the PCA analysis of sample at their initial pH values.

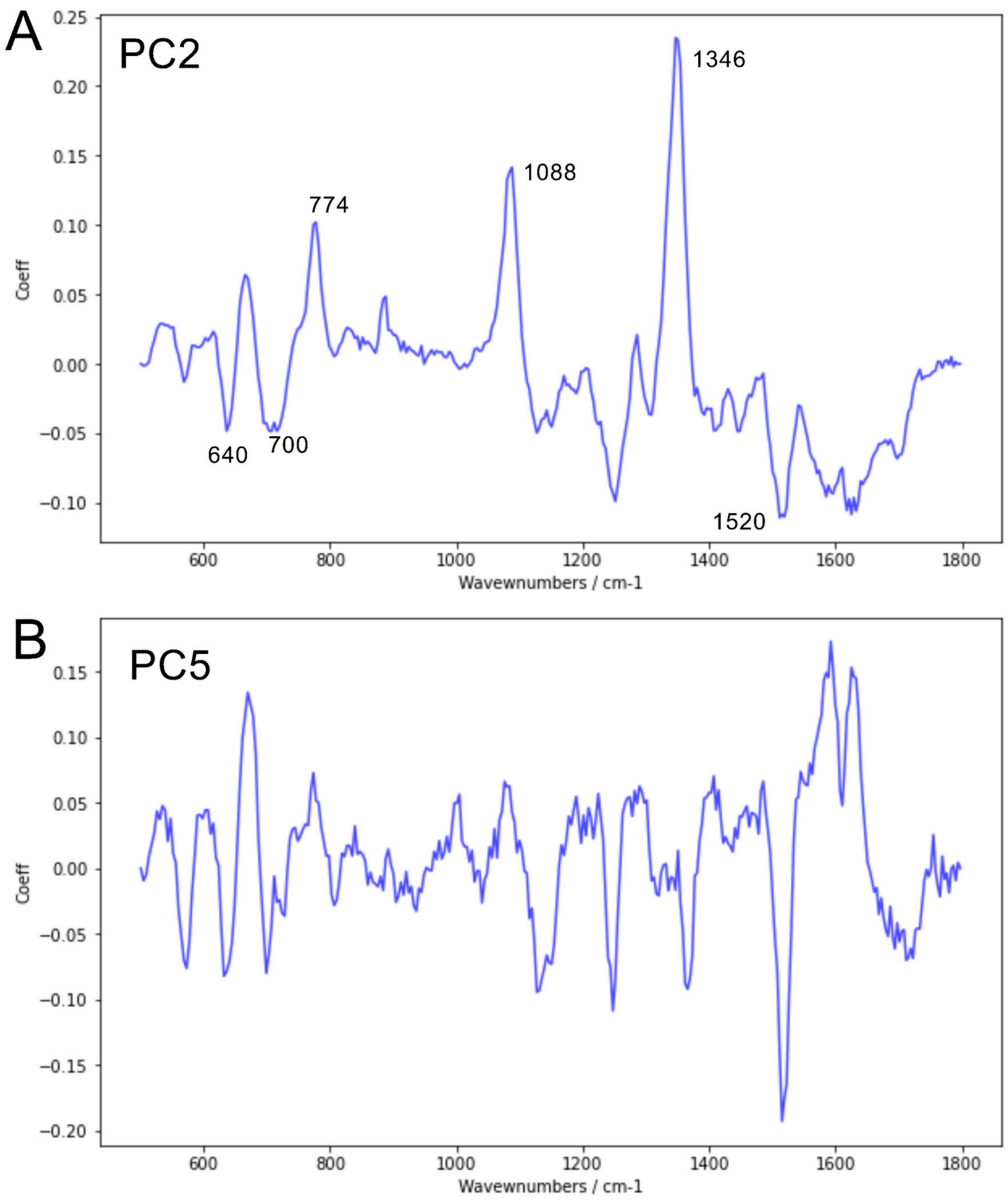

Figure S4. Loading plot of Principal Component 1 (A) and PC 2 (B) from the PCA analysis of sample at pH 5.

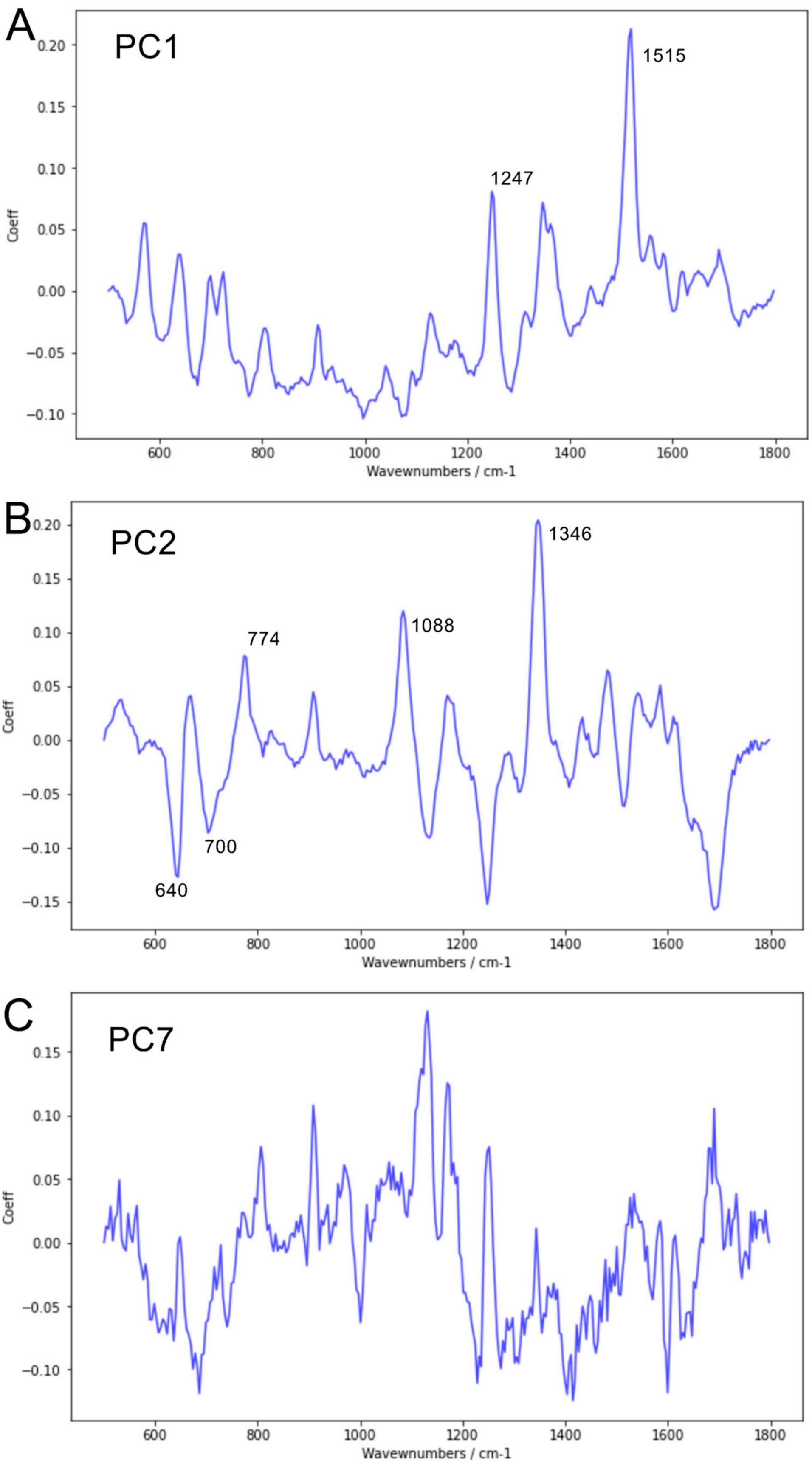

Figure S5. Loading plot of Principal Component 1 (A), PC 2 (B) and PC7 ()C from the PCA analysis of sample at pH 7.

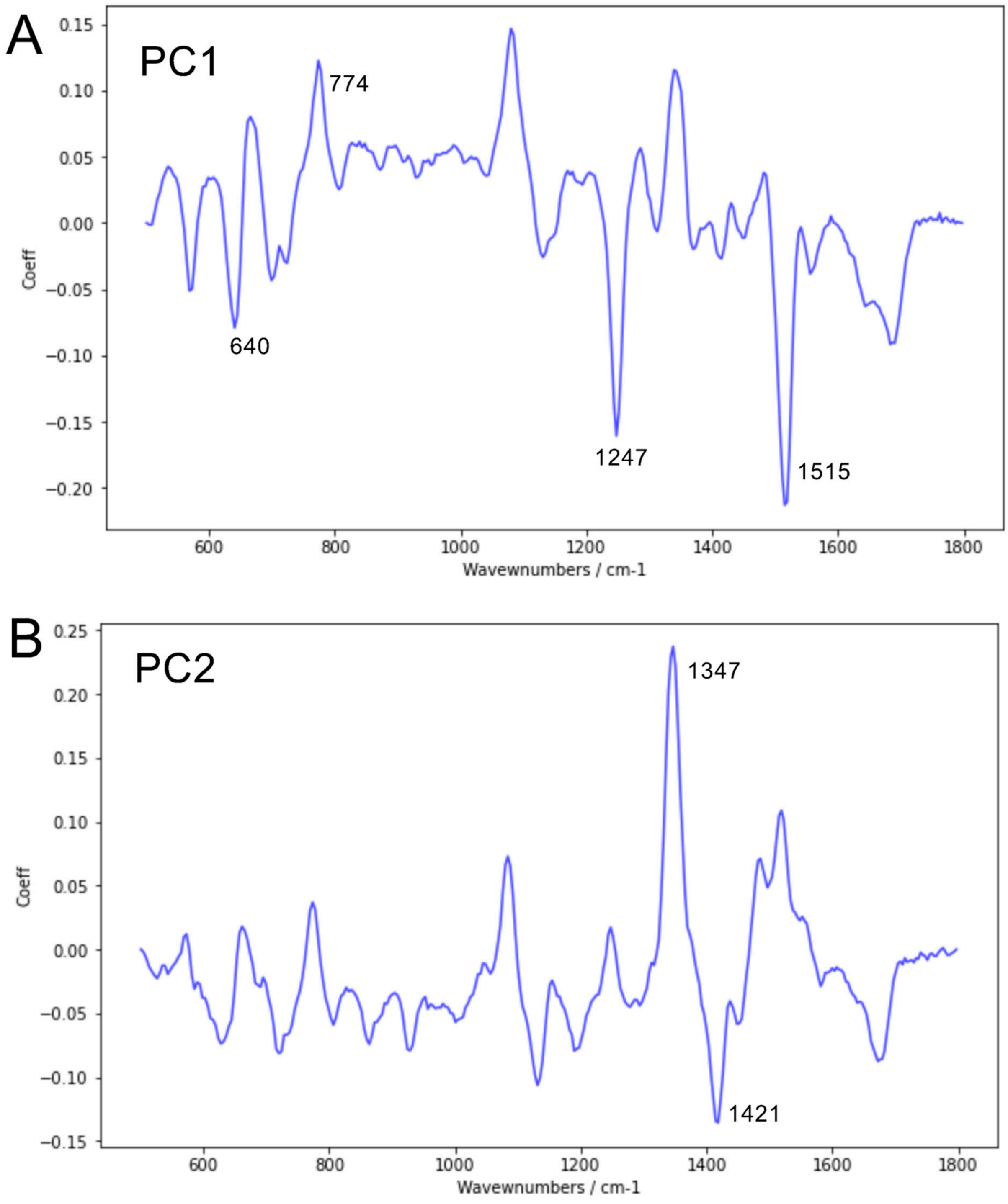

Figure S6. Loading plot of Principal Component 1 (A) and PC 2 (B) from the PCA analysis of sample at pH 9.

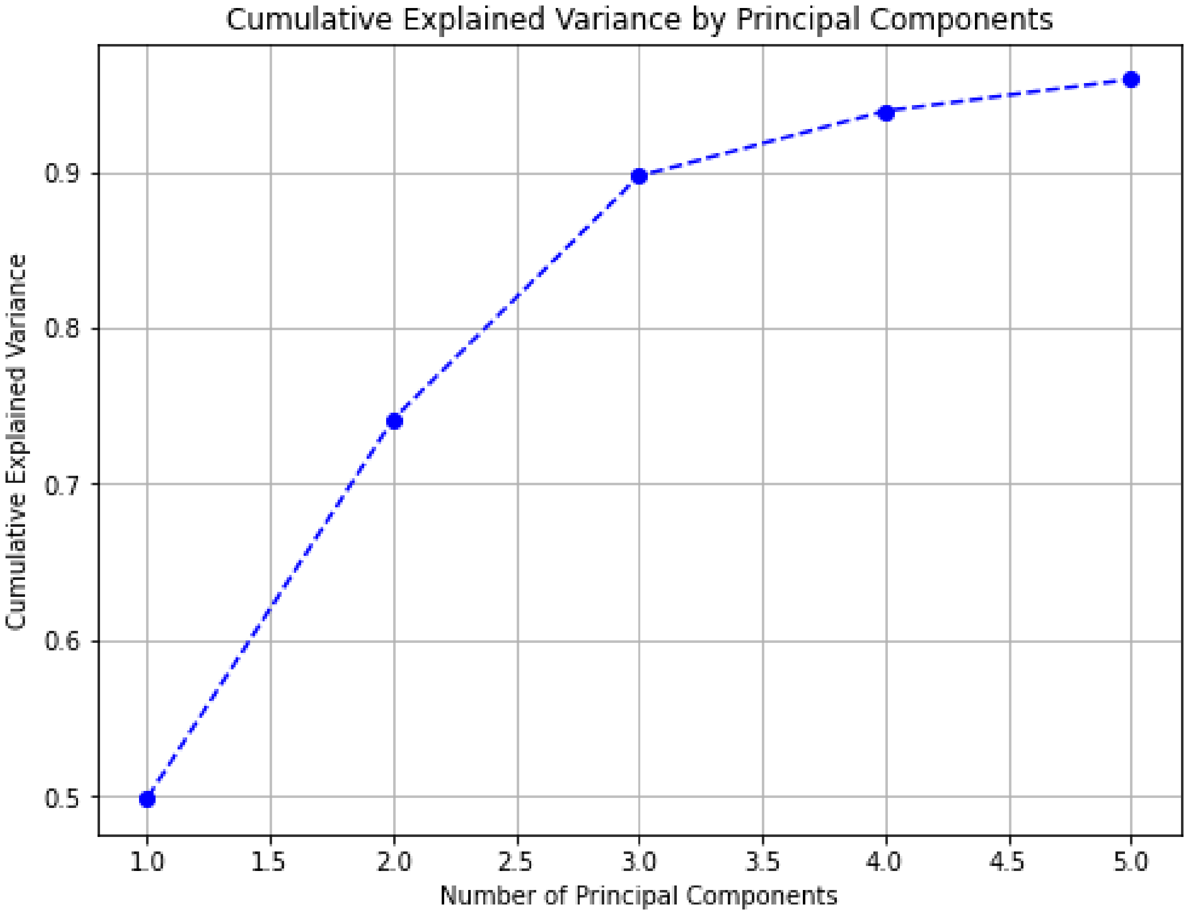

Figure S7. Cumulative explained variance for samples at pH 9 normalized to creatinine.

Classification of samples. To quantify spectral differences, the principal components (PCs) explaining 95% of the variance were used as input for a random forest (RF) classifier. The overall classification accuracy (CA) across all pH values averaged 83.38%, with the highest CA of 89.3% achieved at pH 9. This superior performance at pH 9 is attributed to enhanced molecular information, enabling improved differentiation between CTRL and BC samples.

These findings indicate that pH adjustment, particularly to pH 9, significantly improves the reliability of SERS-based breast cancer detection by optimizing spectral resolution and clustering accuracy. Further investigations are necessary to validate these results and to explore the broader potential of SERS for clinical diagnostic applications.

Table S1. Classification performance of the Logistic Regression classifier for control and breast cancer samples, evaluated using SERS spectra of urine measured at native pH and adjusted pH levels of 5, 7, and 9.

Sample	Classification accuracy (%)	Sensitivity (%)	Specificity (%)
native samples	85.7	85.7	87.6
pH 5 adjusted	78.3	78.3	76.4
pH 7 adjusted	84.6	84.6	86.6
pH 9 adjusted	89.3	89.3	80.7
pH 9 normalized to creatinine	85.7	85.7	83.2
